# Supplementary figures and images for: Eco-morphological diversity of larvae of soldier flies and their closest relatives in deep time
Source: PeerJ. 2020 Nov 27;8:e10356. doi: 10.7717/peerj.10356 (PMC7706506; doi:10.7717/peerj.10356)

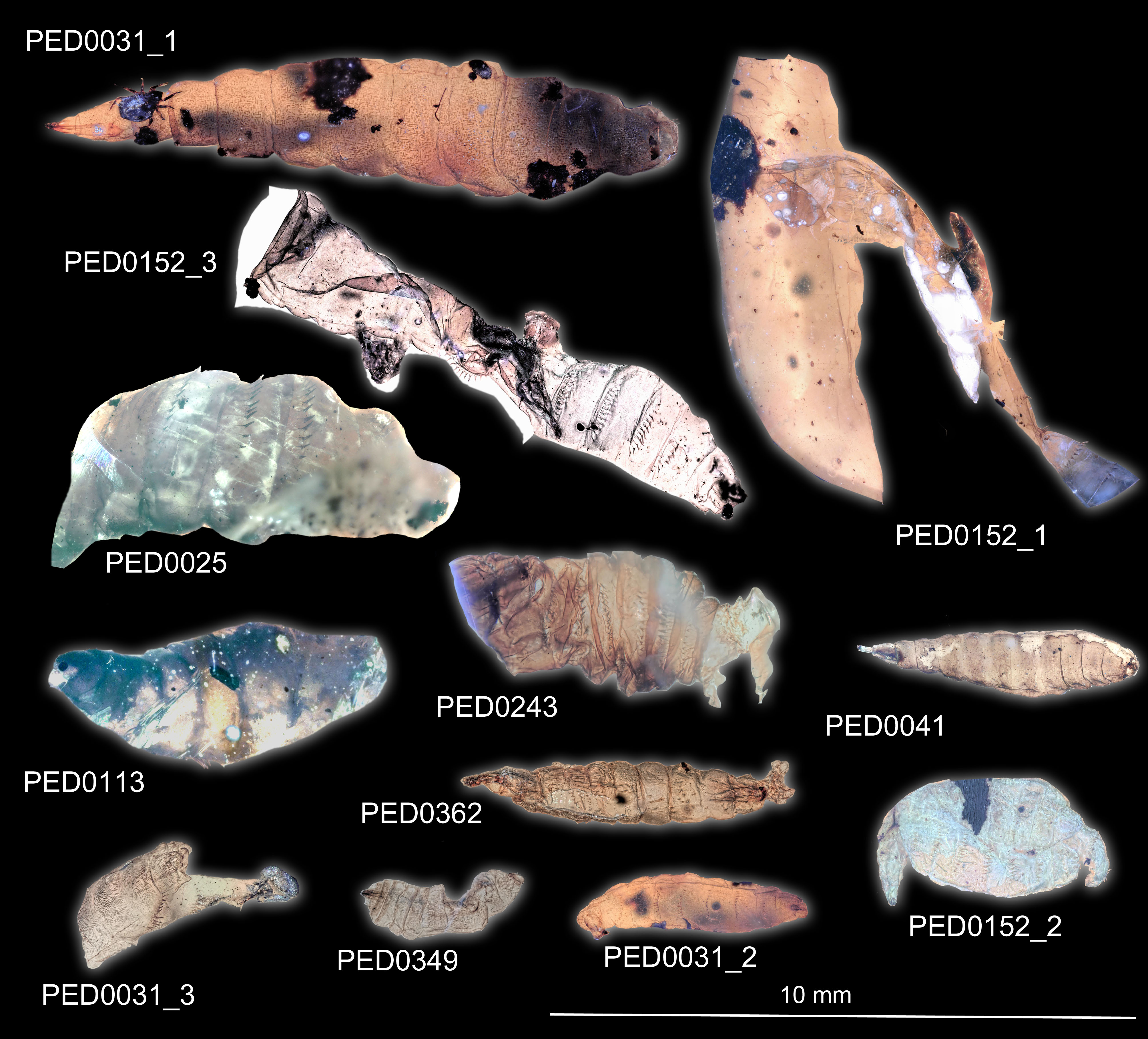

Supplement: Supplemental Information 4 — All specimens are for scale. [file peerj-08-10356-s004.jpg]

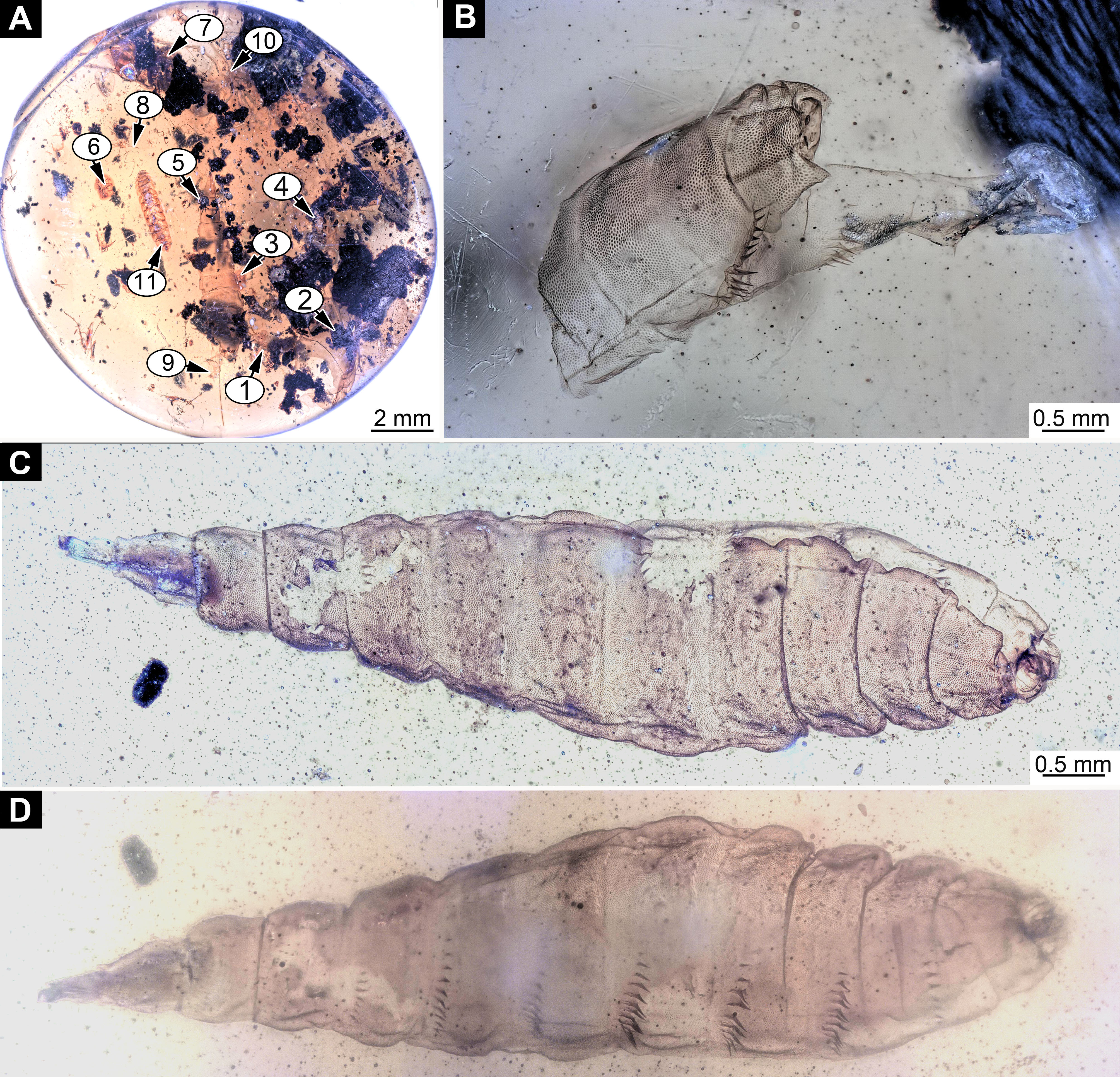

Supplement: Supplemental Information 5 — A) Amber piece PED-0031; B) Morphotype 1 larvae (PED-0031-3) Morphotype 1 larvae (Amber piece PED-0031); C) Morphotype 1 larvae (amber piece PED-0041); D) Morphotype 1 larvae (PED-0041), larva, habitus, ventro-lateral.ventrolateral. Legend: 1–beetle larva, 2–possible cuticle of the morphotype 1 larva, 3–morphotype larva, 4–morphotype 1 larva, 5–mite, 6–possible scale insect, 7-partial– part of a representative of Insecta inclusion, 8 -– fly of the group Bibionomorpha, 9 – beetle larva, 10- Aranea – spider, 11- Myriapoda. – millipede. [file peerj-08-10356-s005.jpg]

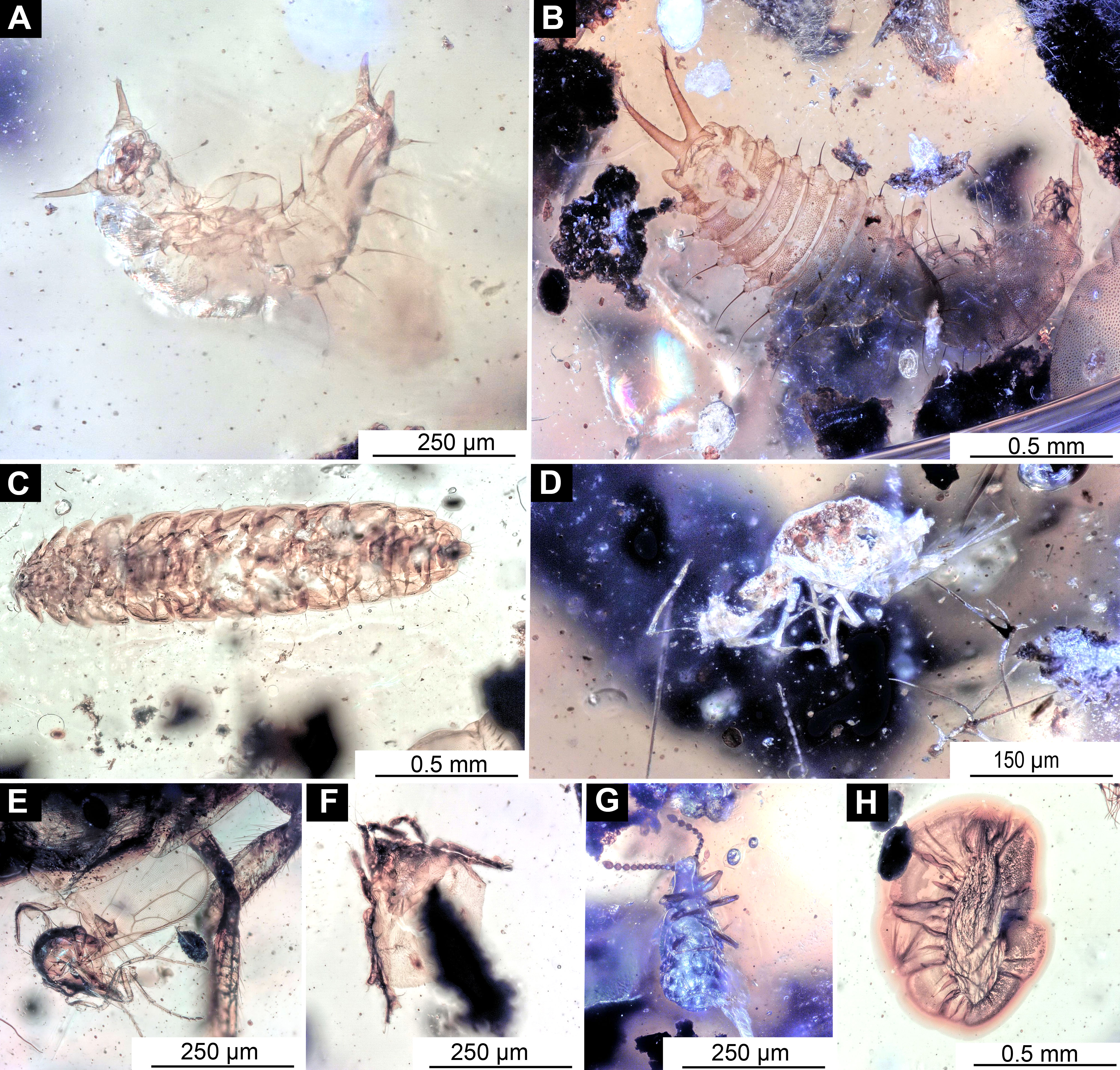

Supplement: Supplemental Information 6 — A-B) Coleoptera representative larvae; C) Myriapoda; D) Possible representative of Collembola; E) Bibonomorpha fly of the group Bibionomorpha; F) Mite; G) Coleoptera,beetle adult; H) Possiblepossible scale insect. [file peerj-08-10356-s006.jpg]

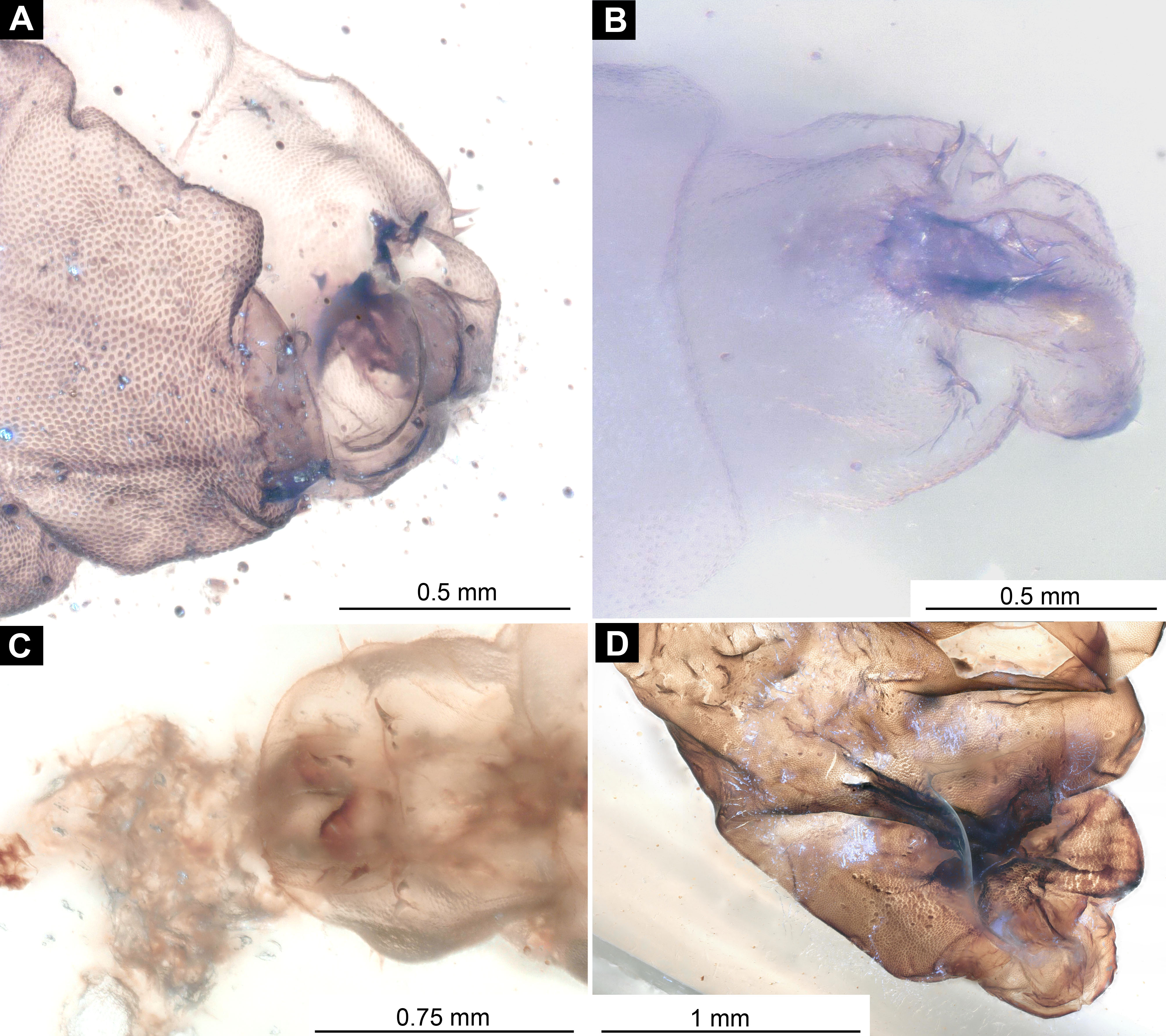

Supplement: Supplemental Information 7 — A) Specimen PED-0041; B) Specimen PED-0031-2; C) Specimen PED-0362 ; D) Specimen PED-0243. [file peerj-08-10356-s007.jpg]

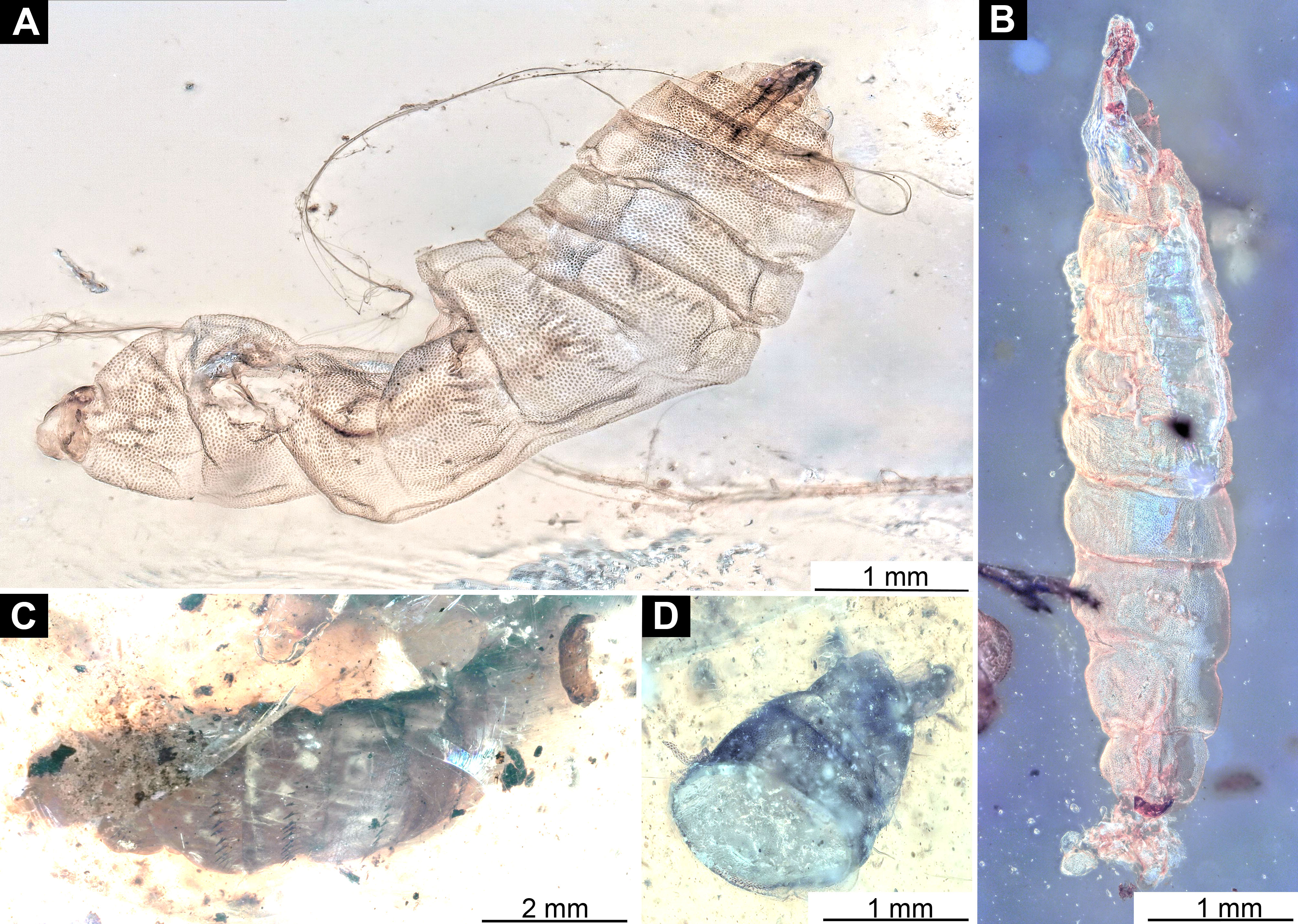

Supplement: Supplemental Information 8 — A) Specimen PED-0349; B) Specimen PED-0362; C) First fragment of the specimen PED-0025; D) Second fragment of the specimen PED-0025. [file peerj-08-10356-s008.jpg]

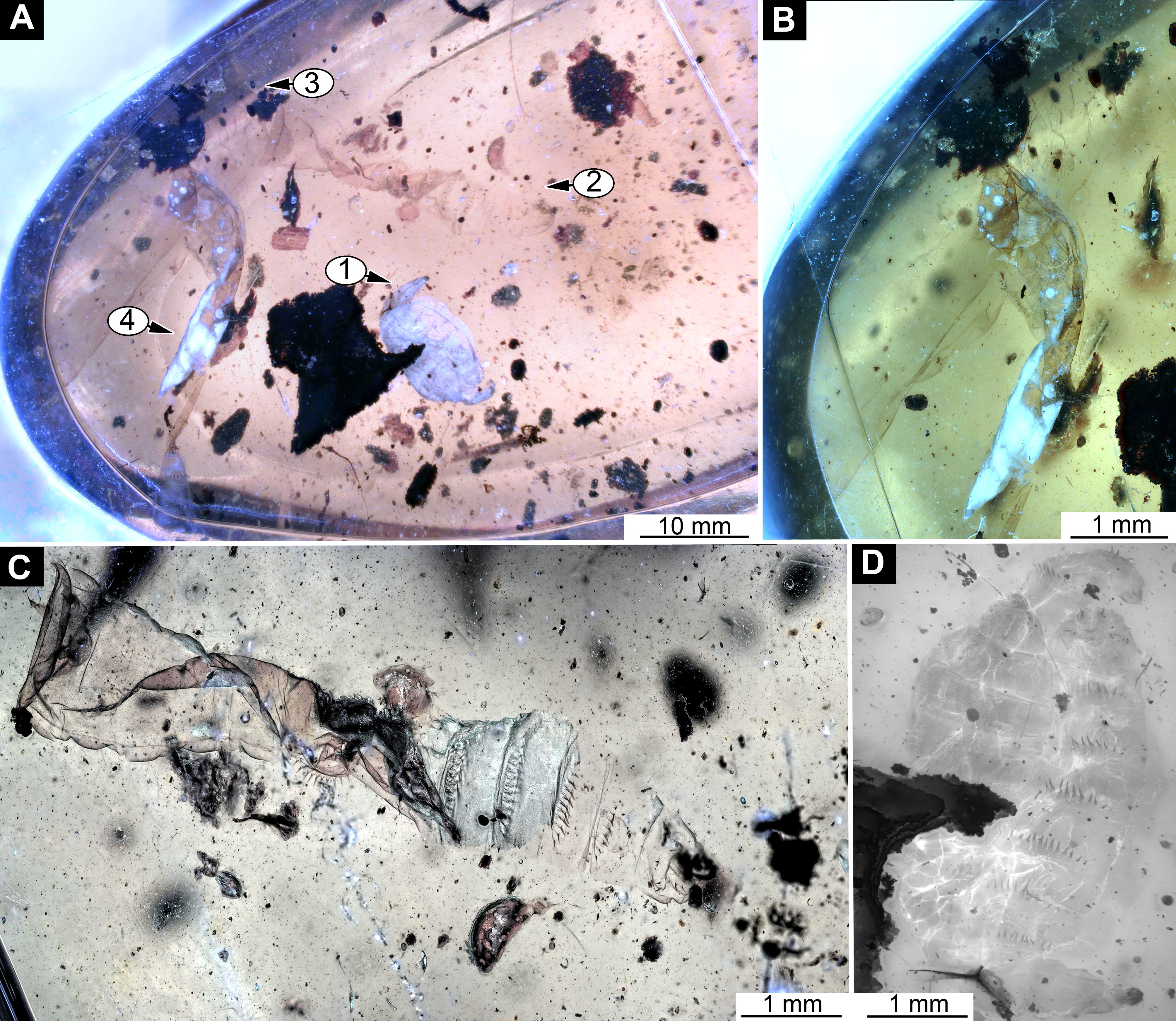

Supplement: Supplemental Information 9 — A) Overview of the amber piece; B-D) Photos of the individual larvae in the piece. Photo D was taken using Keyence BZ-9000 fluorescence microscope. Legend: 1-4 - larvae of the morphotype 1. [file peerj-08-10356-s009.jpg]

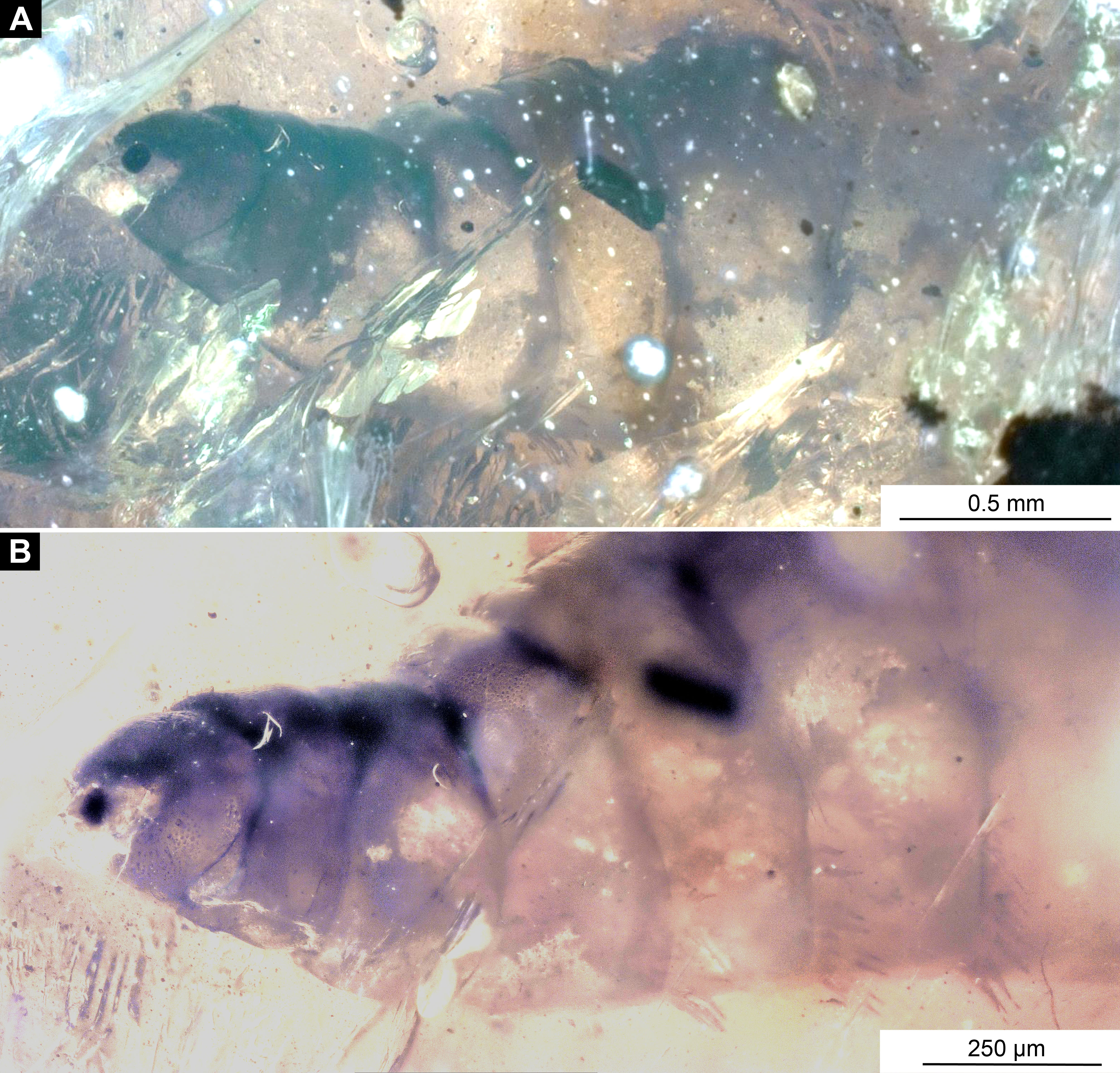

Supplement: Supplemental Information 10 — A) Habitus, overview; B) Trunk’s end, enlarged, with tergal spines clearly visible. [file peerj-08-10356-s010.jpg]

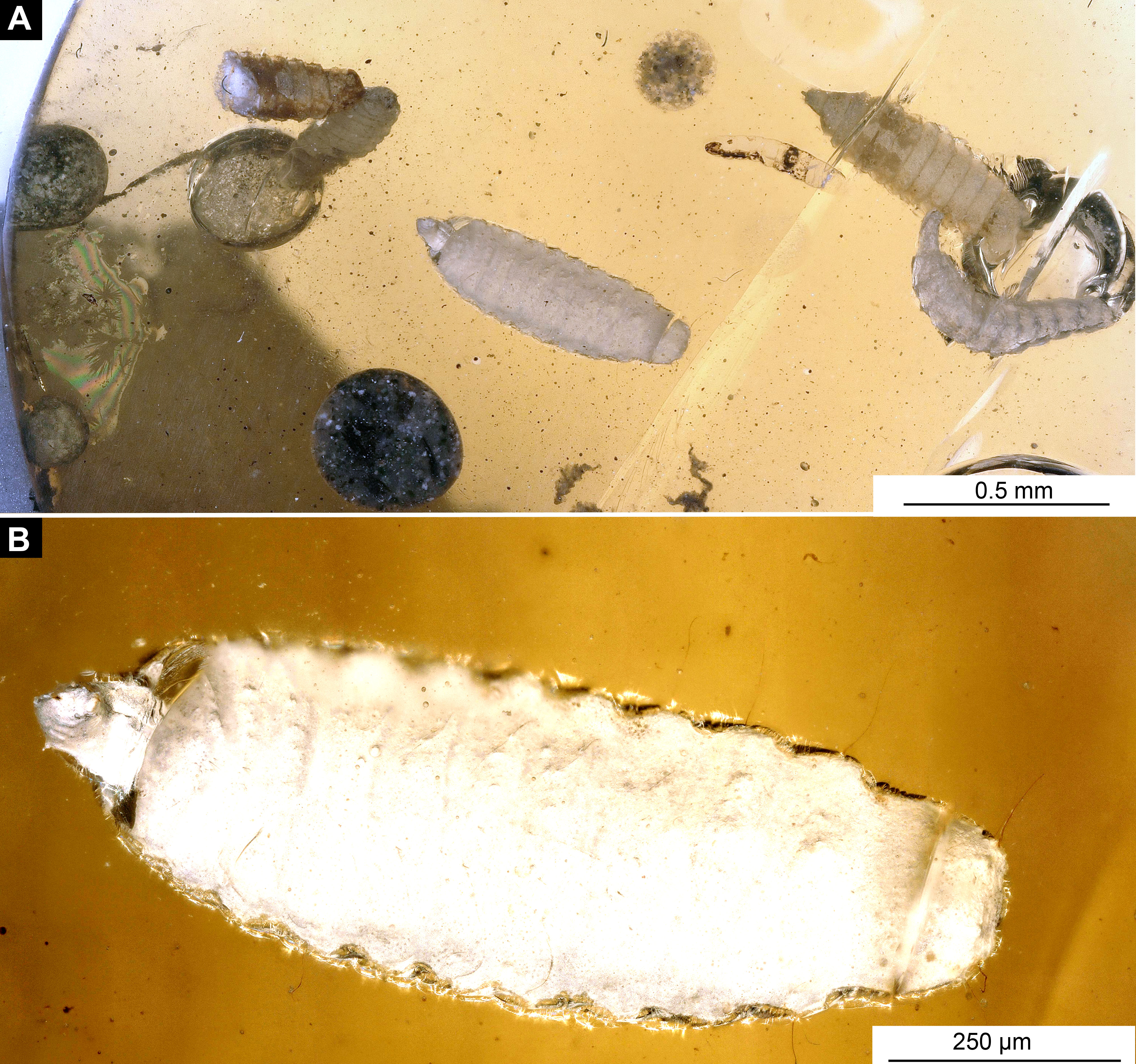

Supplement: Supplemental Information 11 — A) Overview of the amber piece LACM ENT 366281, with 5 larvae; B) Habitus, ventral view of the morphotype 2 larva from the LACM ENT 366281. [file peerj-08-10356-s011.jpg]

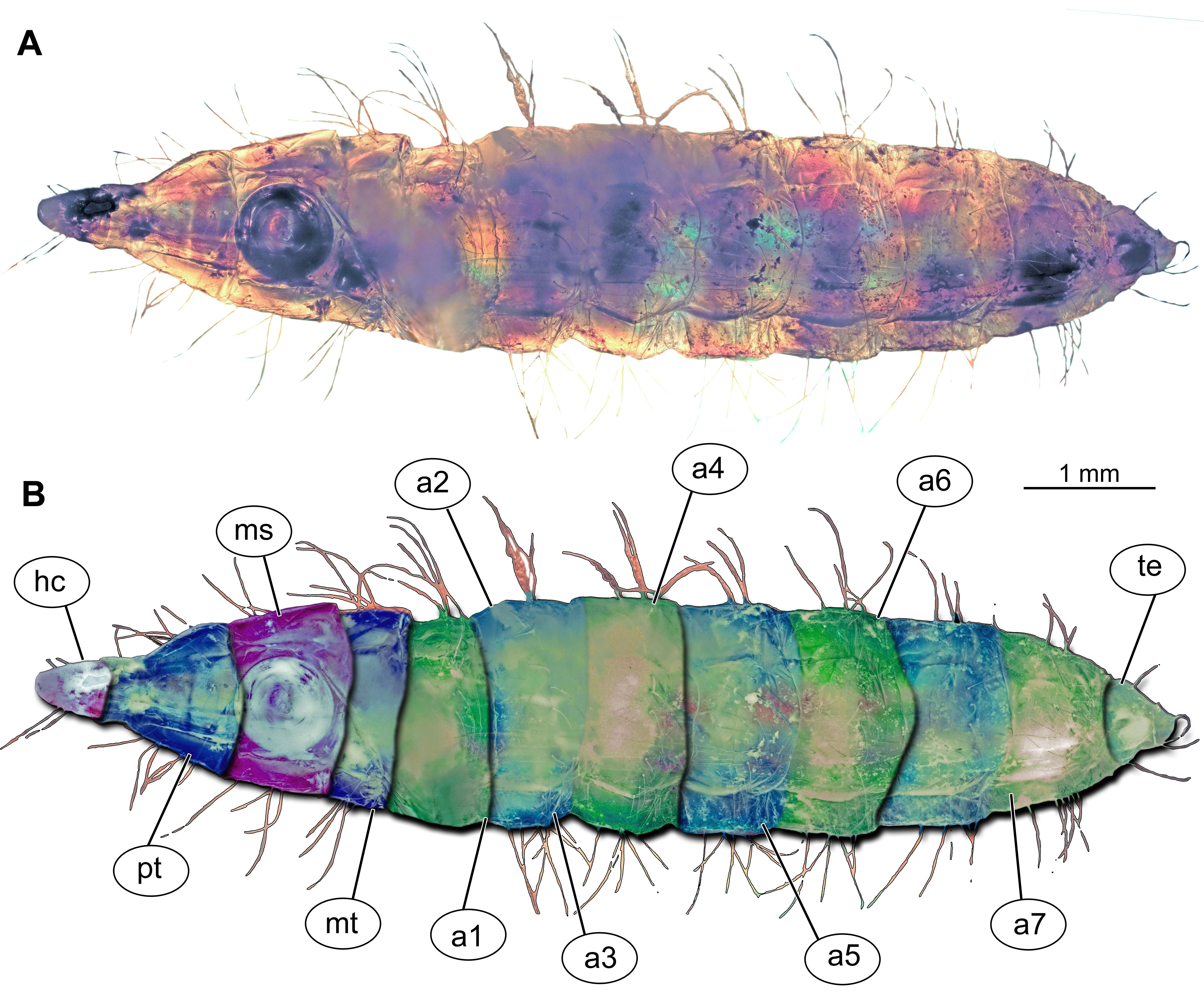

Supplement: Supplemental Information 12 — A) dorsal view; B) dorsal view, marked. Abbreviations: hc- head capsule, pt - prothorax, ms - mesothorax, mt -metathorax, a1-a7 - abdominal units 1-7, te - trunk’s end. [file peerj-08-10356-s012.jpg]

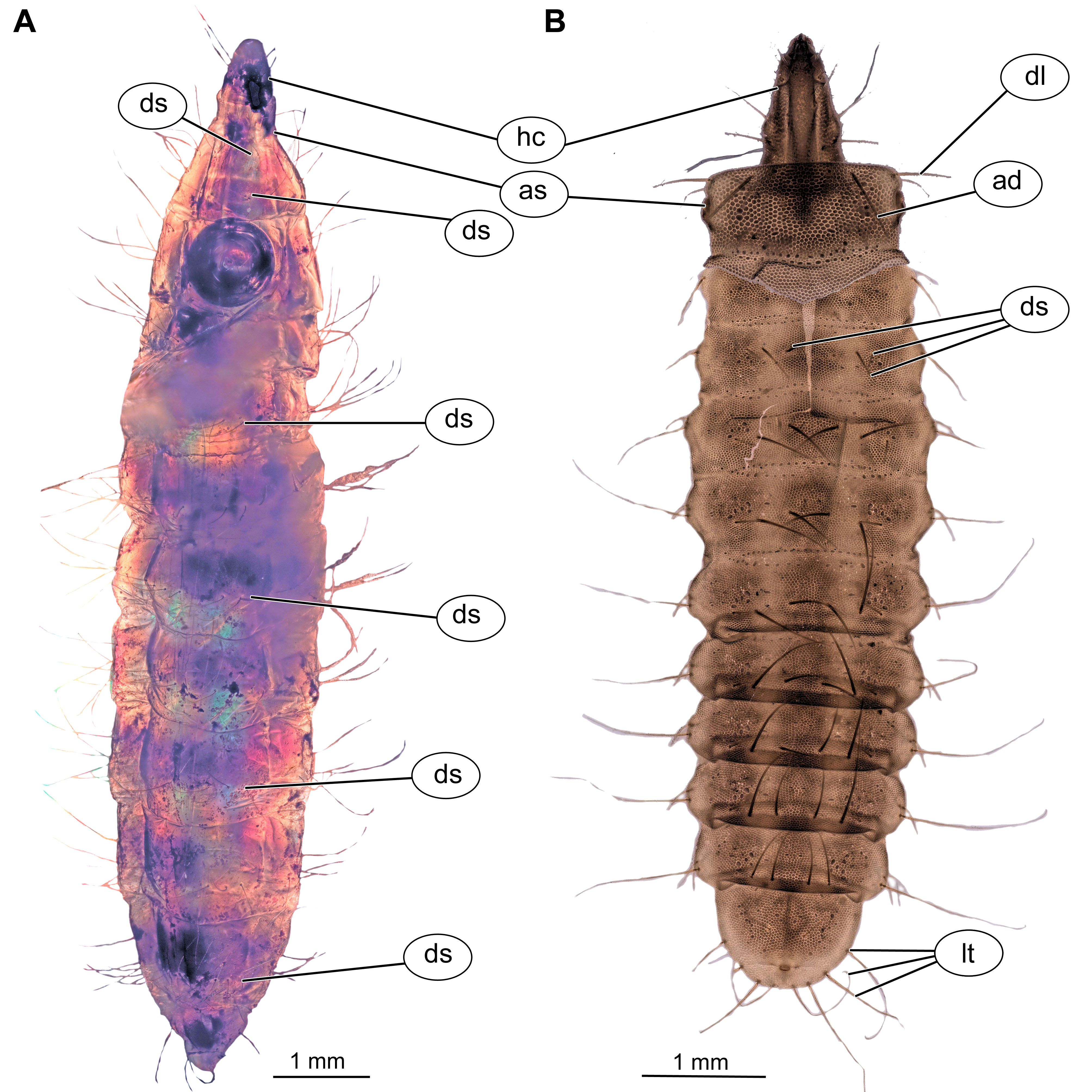

Supplement: Supplemental Information 13 — Abbreviations: hc- head capsule; as - anterior spiracle; ds-dorsal setae; ad -antero-dorsal setae; lt-lateral setae. [file peerj-08-10356-s013.jpg]

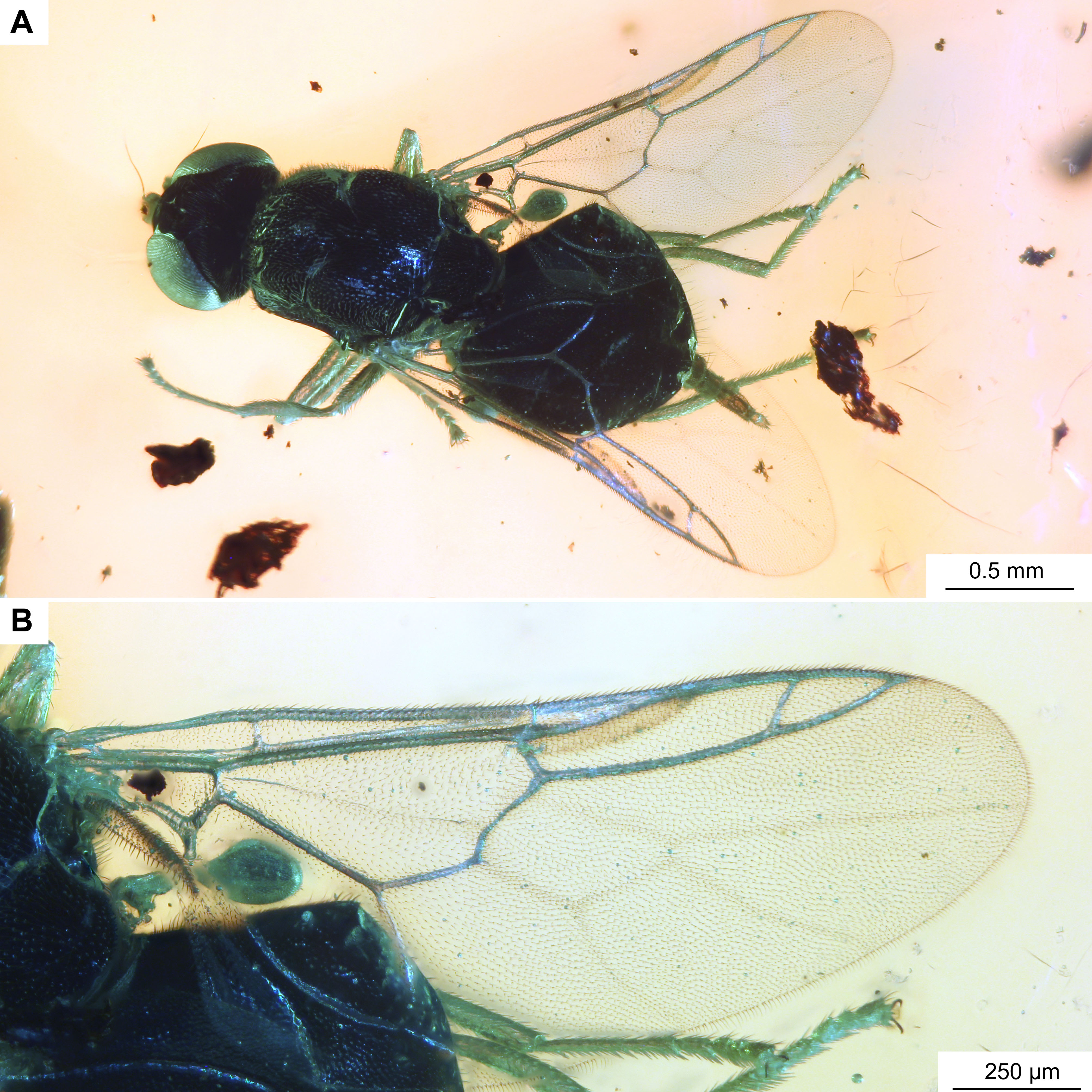

Supplement: Supplemental Information 14 — A) Habitus; B) Close-up photo of wing. [file peerj-08-10356-s014.jpg]

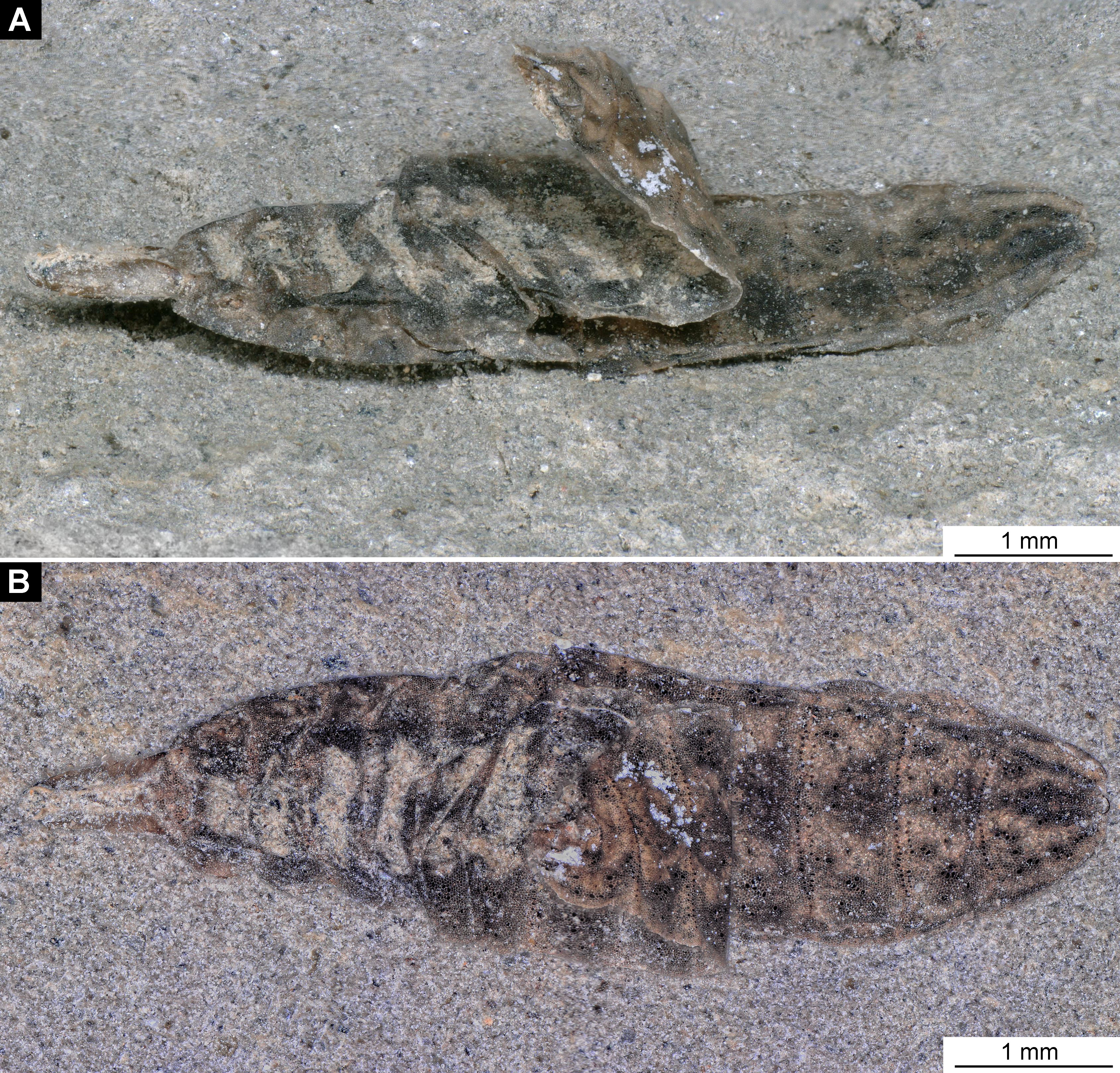

Supplement: Supplemental Information 15 — A) habitus, ventro-lateral view, right-hand side; B) habitus, ventro-lateral view, left-hand side. [file peerj-08-10356-s015.jpg]

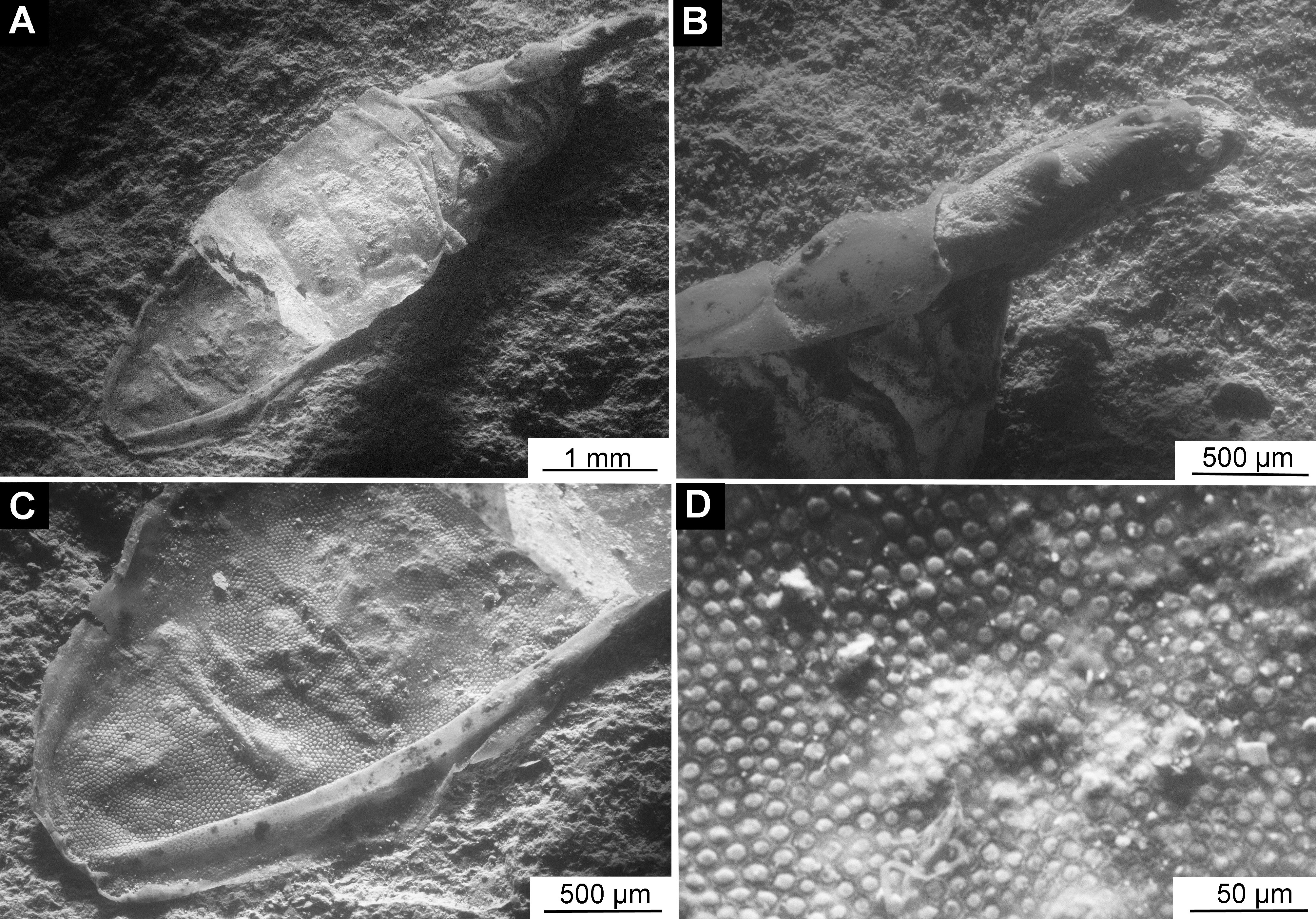

Supplement: Supplemental Information 16 — A). Habitus, ventro-lateral view; B) Head, ventrally; C) Posterior trunk, ventrally; D) Cuticle with Calcium carbonate pallets up close. [file peerj-08-10356-s016.jpg]

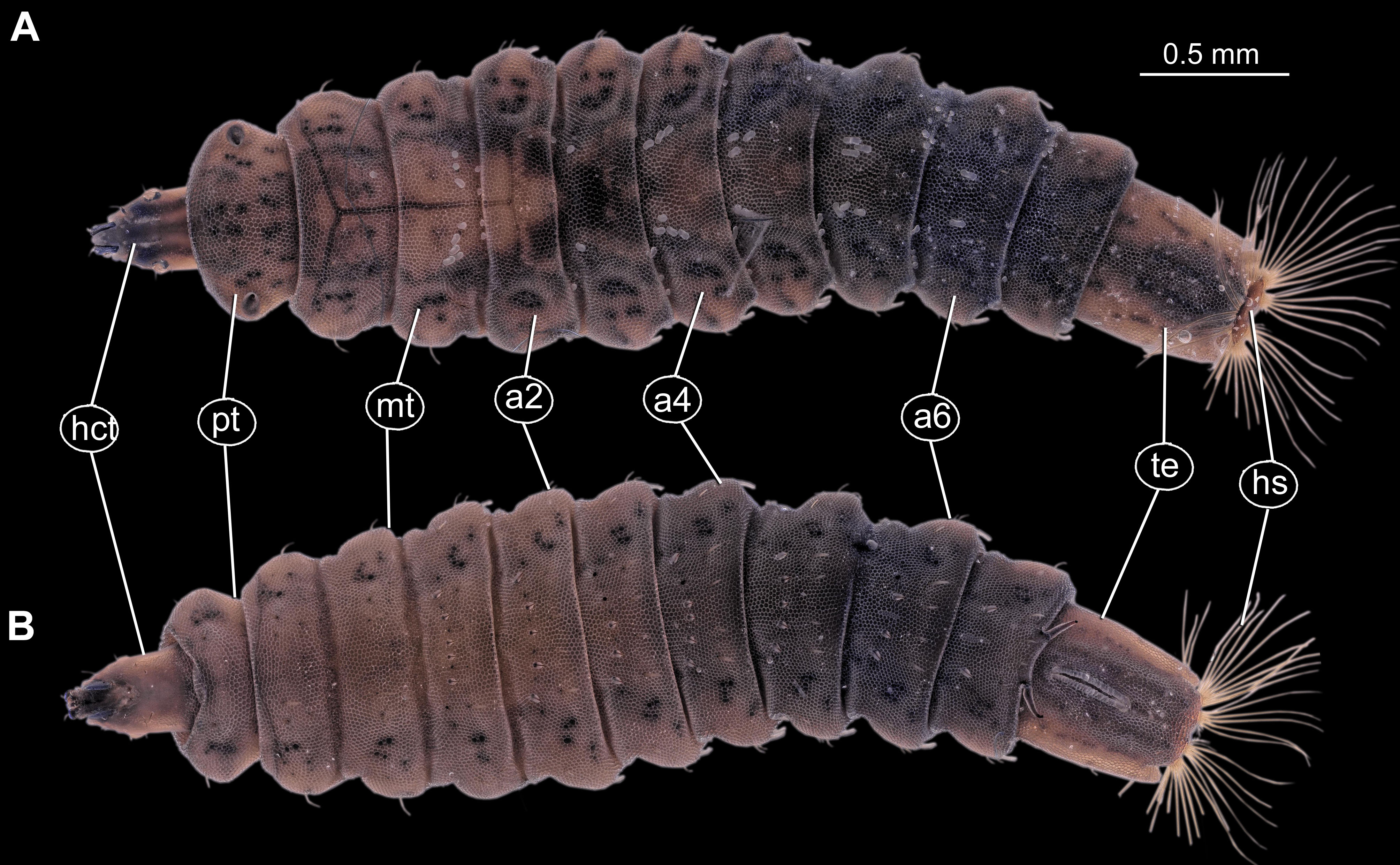

Supplement: Supplemental Information 17 — A) Dorsal view; B) ventral view. Abbreviations: hc-head capsule, pt-prothorax; mt- metathorax; a1-a6 - abdominal units 1-6; te- trunk end. [file peerj-08-10356-s017.jpg]

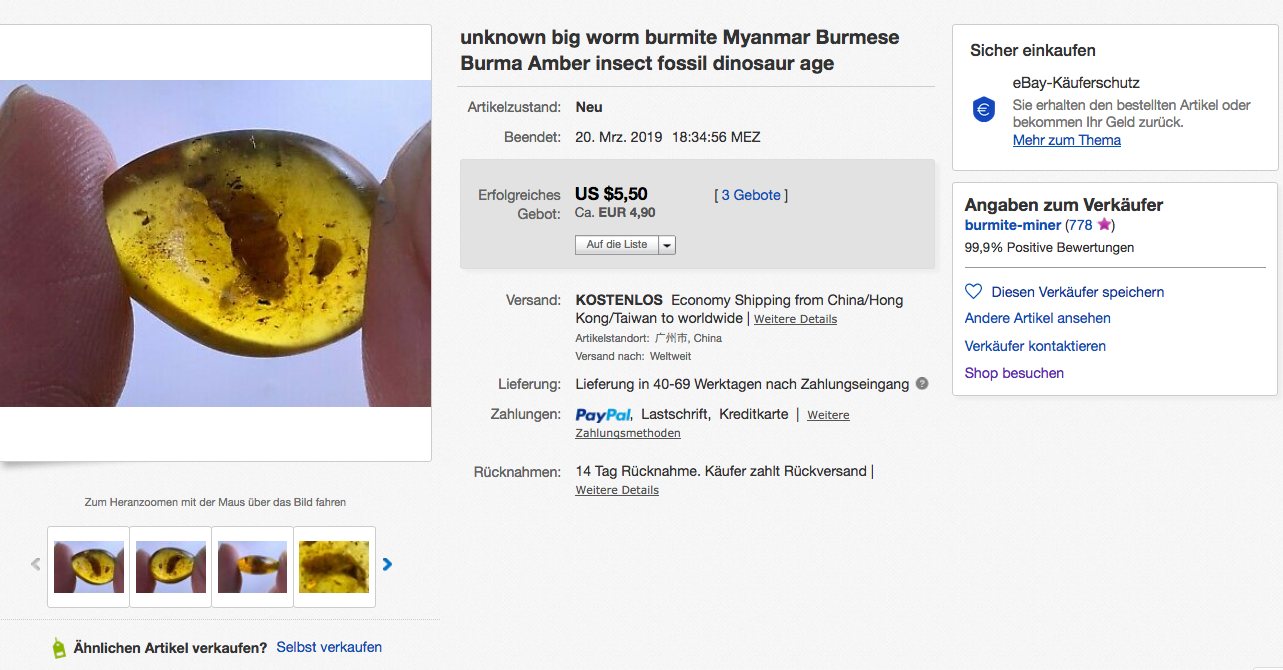

Supplement: Supplemental Information 18 [file peerj-08-10356-s018.png]

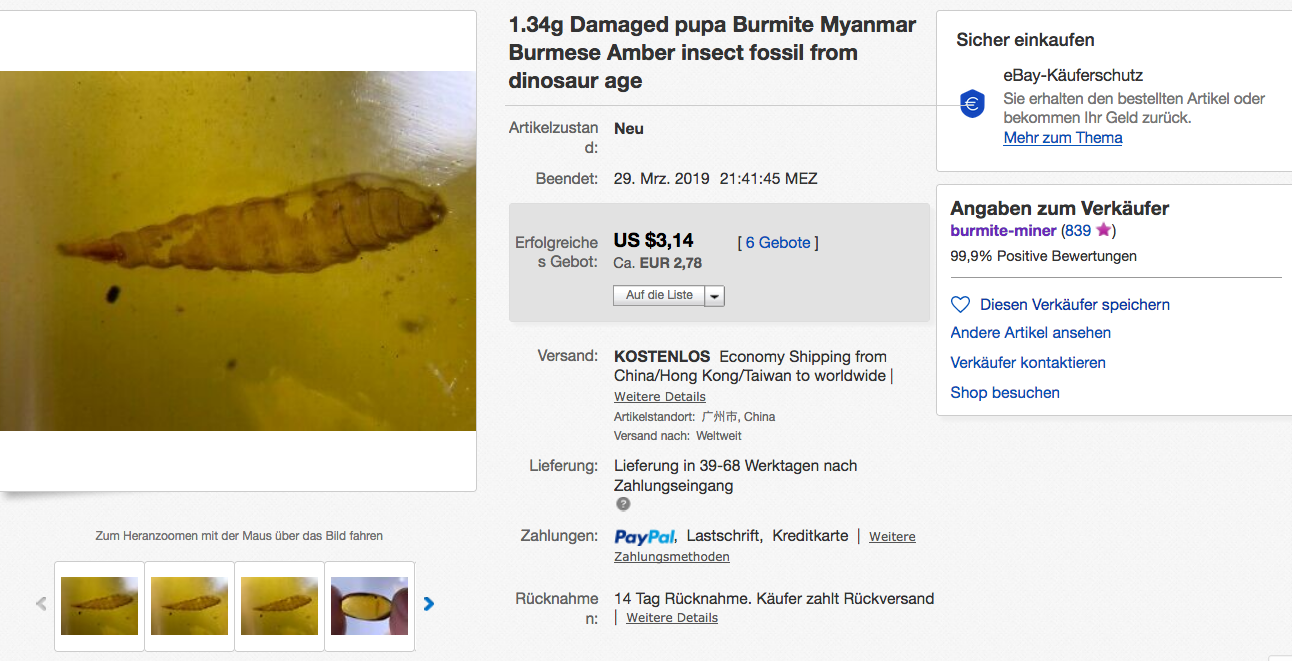

Supplement: Supplemental Information 19 [file peerj-08-10356-s019.png]

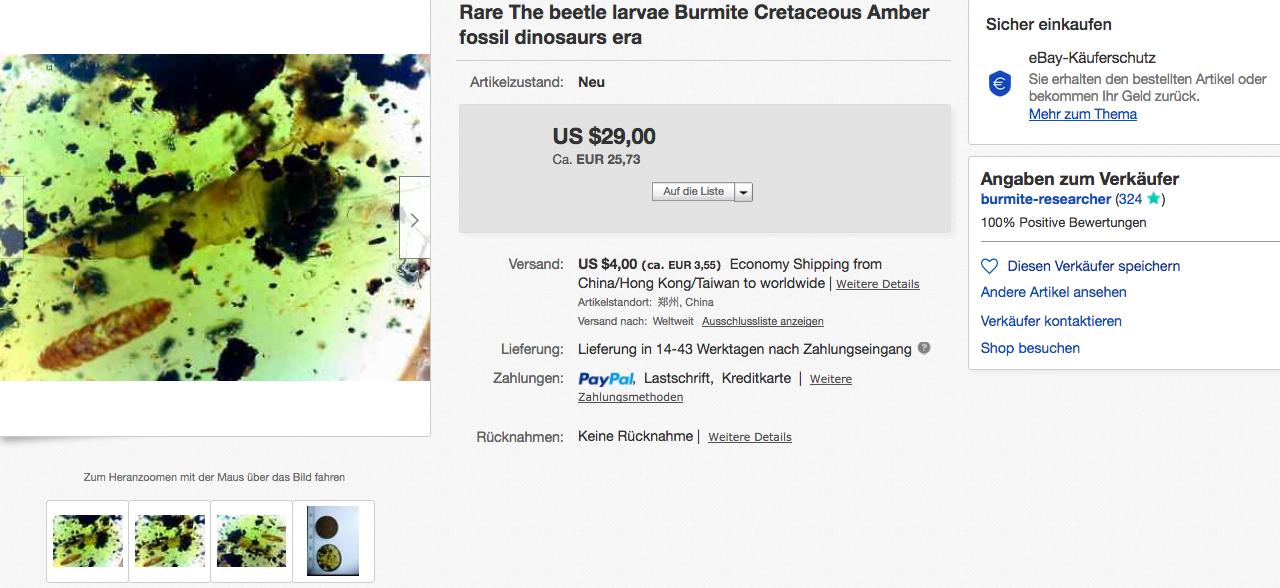

Supplement: Supplemental Information 20 [file peerj-08-10356-s020.png]

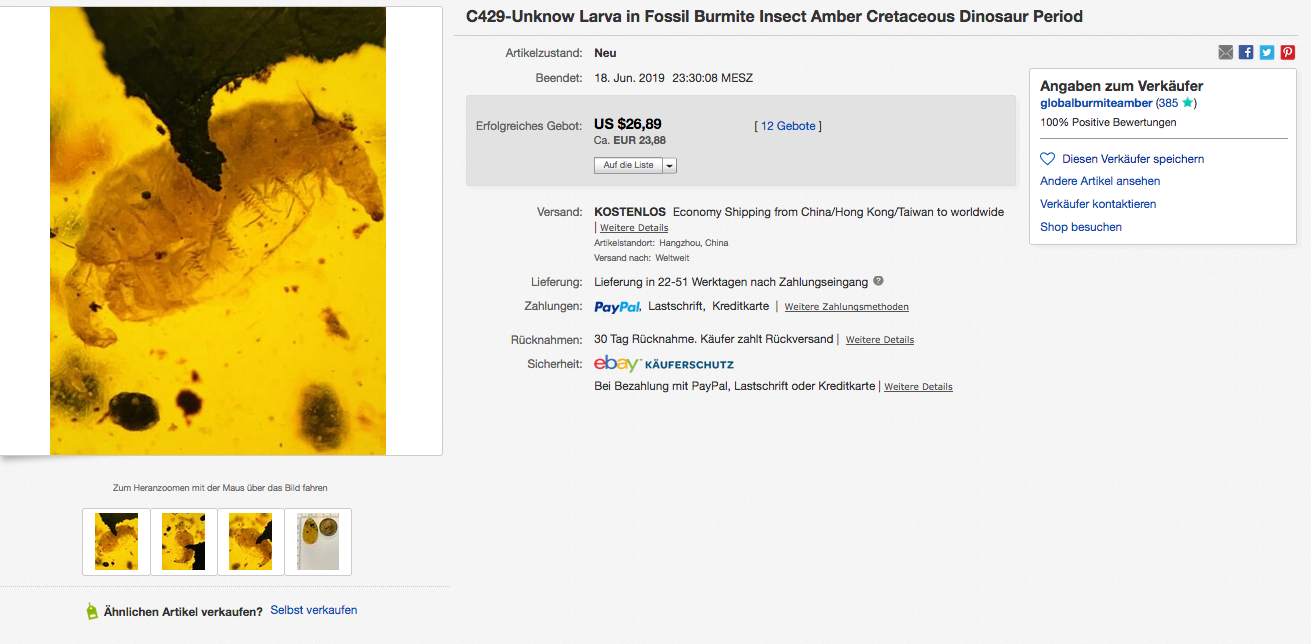

Supplement: Supplemental Information 21 [file peerj-08-10356-s021.png]

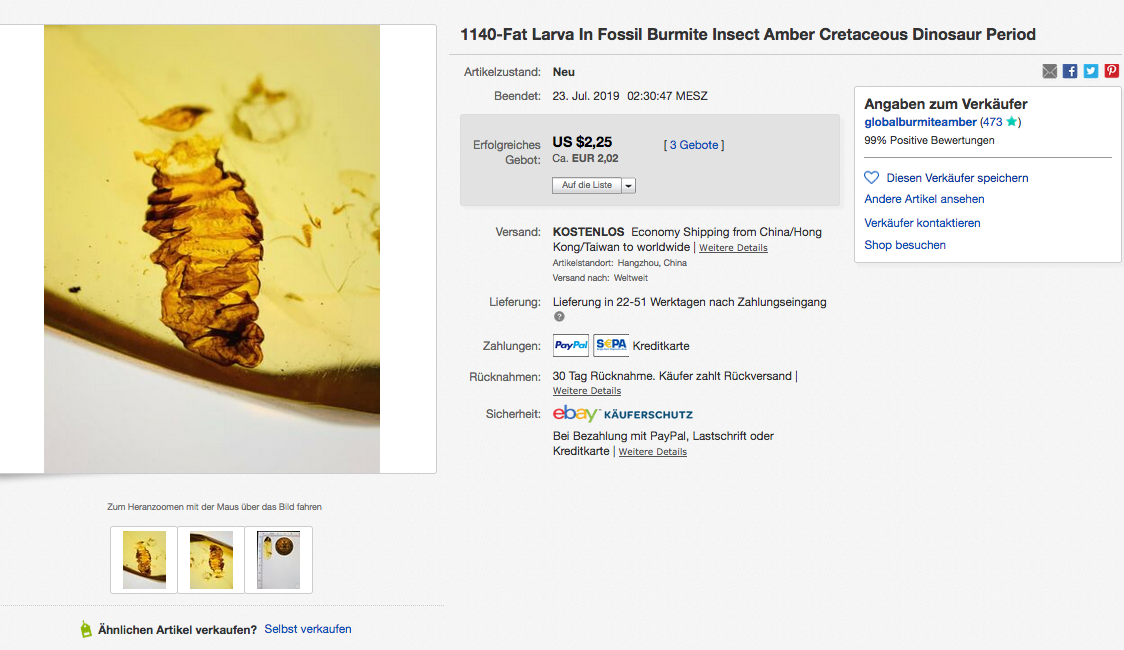

Supplement: Supplemental Information 22 [file peerj-08-10356-s022.png]

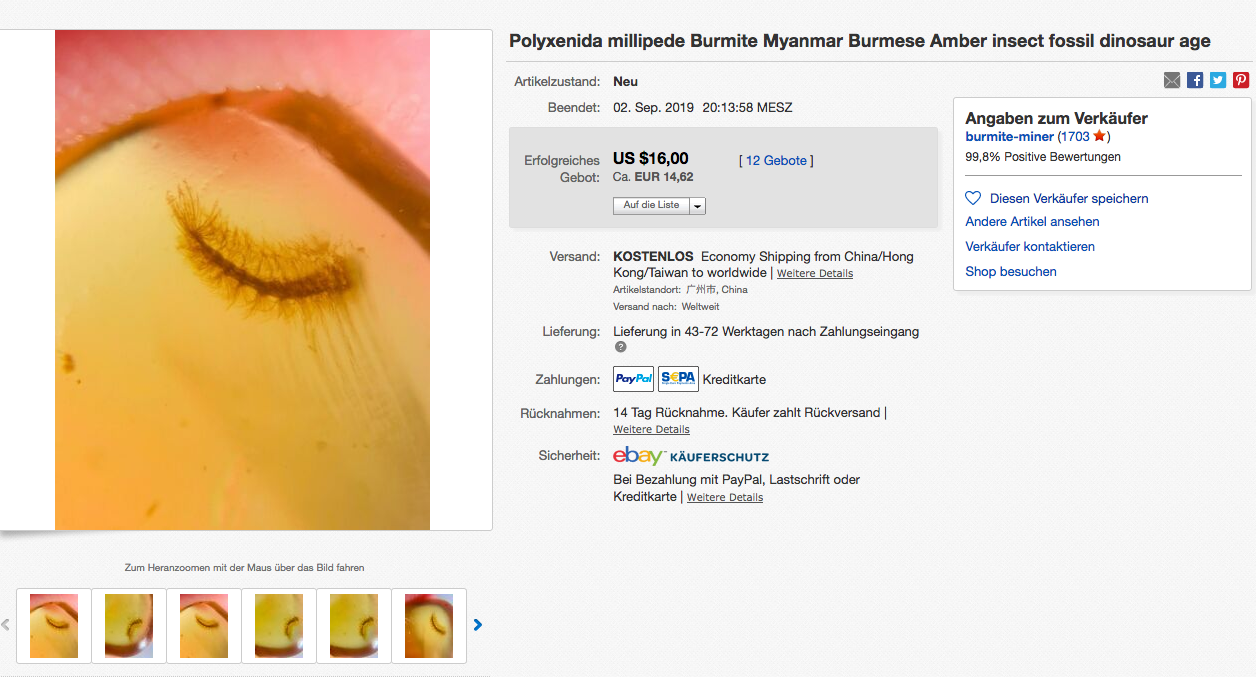

Supplement: Supplemental Information 23 [file peerj-08-10356-s023.png]

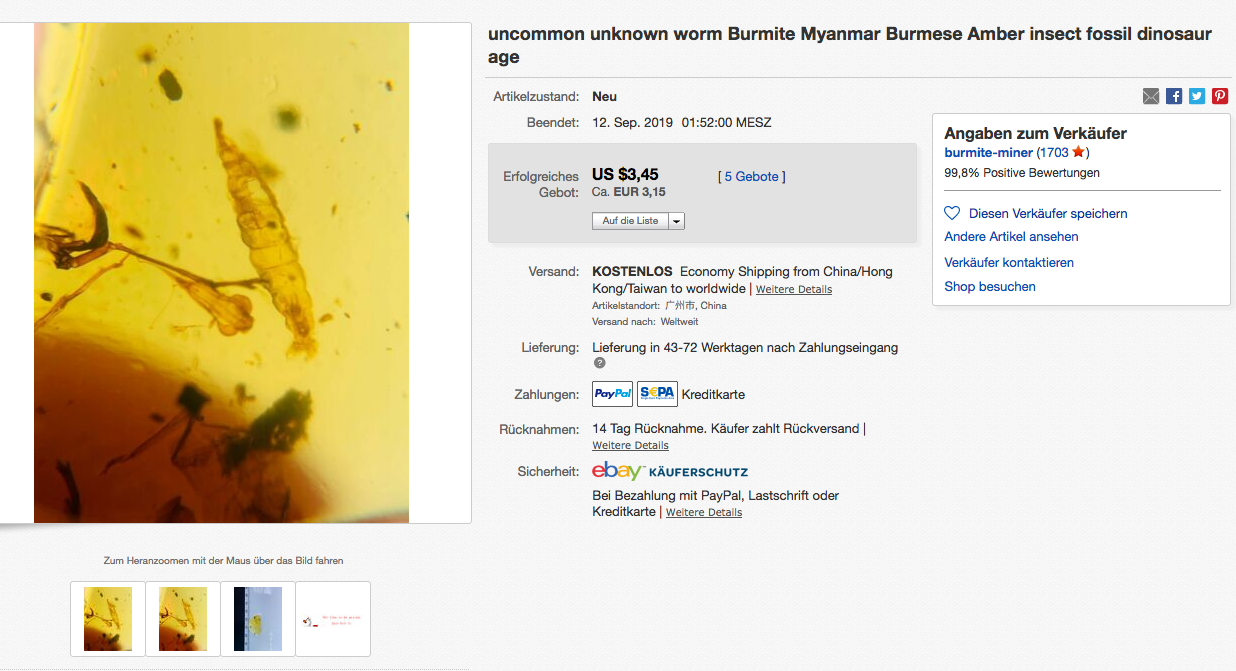

Supplement: Supplemental Information 24 [file peerj-08-10356-s024.png]

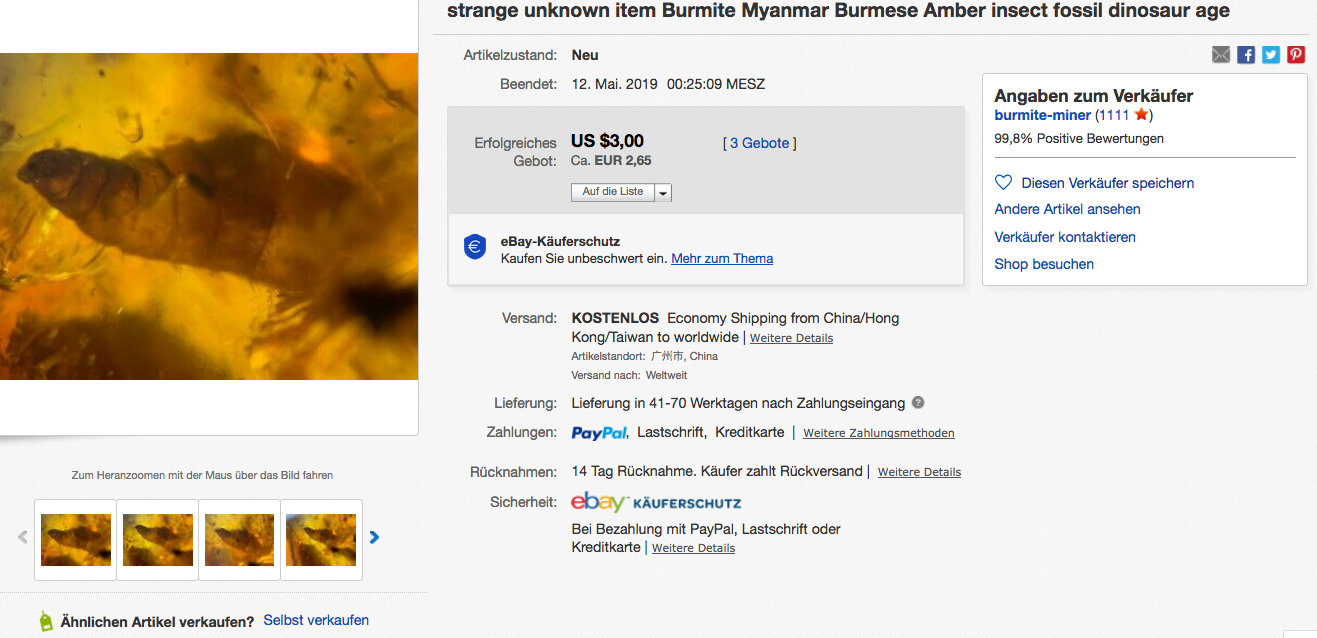

Supplement: Supplemental Information 25 [file peerj-08-10356-s025.png]

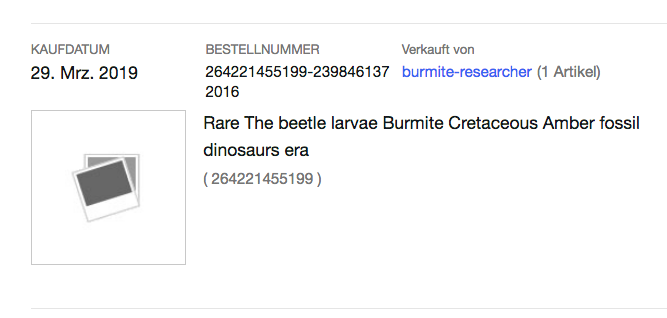

Supplement: Supplemental Information 26 [file peerj-08-10356-s026.png]
